# Seasonality of Planktonic Freshwater Ciliates: Are Analyses Based on V9 Regions of the 18S rRNA Gene Correlated With Morphospecies Counts?

**DOI:** 10.3389/fmicb.2019.00248

**Published:** 2019-02-19

**Authors:** Gianna Pitsch, Estelle Patricia Bruni, Dominik Forster, Zhishuai Qu, Bettina Sonntag, Thorsten Stoeck, Thomas Posch

**Affiliations:** ^1^Limnological Station, Department of Plant and Microbial Biology, University of Zurich, Kilchberg, Switzerland; ^2^Ecology Group, Technical University of Kaiserslautern, Kaiserslautern, Germany; ^3^Research Department for Limnology, Mondsee, University of Innsbruck, Mondsee, Austria

**Keywords:** freshwater ciliates, morphospecies counts, V9 region of the SSU rRNA gene, Lake Zurich, seasonality, planktonic ciliates

## Abstract

Ciliates represent central nodes in freshwater planktonic food webs, and many species show pronounced seasonality, with short-lived maxima of a few dominant taxa while many being rare or ephemeral. These observations are primarily based on morphospecies counting methods, which, however, have limitations concerning the amount and volume of samples that can be processed. For high sampling frequencies at large scales, high throughput sequencing (HTS) of freshwater ciliates seems to be a promising tool. However, several studies reported large discrepancy between species abundance determinations by molecular compared to morphological means. Therefore, we compared ciliate DNA metabarcodes (V9 regions of the 18S rRNA gene) with morphospecies counts for a 3-year study (Lake Zurich, Switzerland; biweekly sampling, *n* = 74). In addition, we isolated, cultivated and sequenced the 18S rRNA gene of twelve selected ciliate species that served as seeds for HTS analyses. This workflow allowed for a detailed comparison of V9 data with microscopic analyses by quantitative protargol staining (QPS). The dynamics of V9 read abundances over the seasonal cycle corroborated well with morphospecies population patterns. Annual successions of rare and ephemeral species were more adequately characterized by V9 reads than by QPS. However, numbers of species specific sequence reads only partly reflected rank orders seen by counts. In contrast, biomass-based assemblage compositions showed higher similarity to V9 read numbers, probably indicating a relation between cell sizes and numbers / sizes of macronuclei (or 18S rRNA operons). Full-length 18S rRNA sequences of ciliates assigned to certain morphospecies are urgently needed for barcoding approaches as planktonic taxa are still poorly represented in public databases and the interpretation of HTS data depends on profound reference sequences. Through linking operational taxonomic units (OTUs) with known morphospecies, we can use the deep knowledge about the autecology of these species.

## Introduction

In temperate lakes, seasonal successions of phyto- and metazooplankton often show predictive dynamics, which were described in a conceptual framework, the Plankton Ecology Group (PEG) model (Sommer et al., 1986). During the last 30 years, ecological research has widened the understanding of mechanisms responsible for seasonality with a key finding that dynamics of heterotrophic protists should be regarded separately from metazoan zooplankton patterns (Sommer et al., 2012). Microzooplankton may account for a major proportion of total grazing on phytoplankton, consuming more than 50% of primary production during distinct periods in the year (Weisse et al., 1990; Straile, 1998). The algivorous protist fraction is often dominated by ciliates (Ciliophora), representing, e.g., the first and most effective grazers of phytoplankton spring blooms (Geller et al., 1991; Sommaruga and Psenner, 1993; Tirok and Gaedke, 2007; Posch et al., 2015). However, feeding modes of ciliate species are quite diverse (bacterivores, algivorous, omnivorous, predacious), thus, they form a major fraction of protistan plankton throughout the year (Mathes and Arndt, 1995; Straile, 1998). Ciliates may even be the dominant bacterivores (Kisand and Zingel, 2000; Zingel et al., 2007) or consumers of autotrophic picoplankton (Šimek et al., 1995) in certain lakes. Seasonal successions of ciliate assemblages and therein involved species have been well described for various temperate lakes (Beaver and Crisman, 1989; Müller et al., 1991; Carrias et al., 1998; Sonntag et al., 2006; Zingel and Nõges, 2010; see also the extensive literature summary in Foissner et al., 1999). These studies have one common thread – their outcome is based on the identification and quantification of morphologically well-defined species (morphospecies, Foissner et al., 1999) via various microscopy techniques. Morphospecies counting allows for the quantification of abundances and biomasses per water volume, which is a prerequisite to study energy flows between trophic entities in lakes.

Since 20 years, the traditional morphology based approaches were either supplemented or even replaced by molecular methods in ecological surveys (Caron et al., 1999; Stoeck et al., 2006; Duff et al., 2008; Berdjeb et al., 2018). Nowadays, high throughput sequencing (HTS) technologies have revolutionized the study of protistan communities in freshwater ecosystems (Simon et al., 2015; Banerji et al., 2018; Bock et al., 2018; Mikhailov et al., 2018). Among others, environmental HTS offers the potential to discover cryptic and rare species which are most likely not recognized by microscopy (Amaral-Zettler et al., 2009; Nolte et al., 2010). Nevertheless, molecular methods for analyzing pro- and eukaryotic microbes have to cope with multiple sources of bias. Above all, PCR is a major source of errors due to primer bias and preferential amplification (Stoeck et al., 2006; Shakya et al., 2013; Tragin et al., 2018; Wear et al., 2018). One major issue emerging from PCR and primer bias is the critical translation of amplicon abundance into organismic abundance (Egge et al., 2013; Weber and Pawlowski, 2013; Stoeck et al., 2014), but for solutions see also Giner et al. (2016). Further sources of error include sequencing (Huse et al., 2007), amplicon clustering (Huse et al., 2010; Forster et al., 2016), and incompleteness and errors in reference databases (Stoeck et al., 2014). Such errors can have extensive consequences for ciliate diversity estimations, often leading to an overestimation of operational taxonomic units (OTUs) richness in an environment. Beyond, many OTUs still remain unassigned and numerous ciliate morphospecies are not yet represented in reference databases (e.g., NCBI). Moreover, the high copy number of 18S rRNA genes in ciliates (Gong et al., 2013) and the fact that copy numbers vary greatly among species, could make quantitative results even more unreliable (Medinger et al., 2010). Thus, it is questionable if HTS approaches alone can be used to answer ecological questions about ciliate species such as succession patterns and their relevance for ecosystem functioning. Several surveys used a combination of molecular and morphological methods to study the diversity and dynamics of planktonic ciliate assemblages, both from marine (Bachy et al., 2013, 2014; Santoferrara et al., 2014, 2016) as well as from freshwater systems (Medinger et al., 2010; Luo et al., 2011; Stoeck et al., 2014). The simultaneous identification of morphospecies besides environmental sequencing helps to differentiate meaningful OTUs from potential sequencing errors. Finally, isolation and subsequent sequencing of taxonomic marker genes from identified ciliate species will enable us to link sequencing data (OTUs) to organisms and their known autecology.

In this study, we conducted a 3-year sampling campaign in Lake Zurich (biweekly sampling, *n* = 74) to establish a substantial data set for the evaluation and improvement of ciliate specific HTS data. Both methods, the classical morphospecies counting via quantitative silver impregnation and 18S rRNA amplicon sequencing, were used to investigate the variability in the ciliate assemblage over time. To test if the two different approaches corroborated with each other, we focused on seasonal patterns of twelve specific ciliate morphospecies. These species were isolated directly from lake water and most of them were successfully cultivated. Subsequently, their V9 regions of the 18S rRNA gene were sequenced and used as seeds for HTS analyses. This workflow resulted in new sequence information for several well-known ciliate morphospecies living in freshwater systems. We intended to address the following aims: (a) Are seasonal patterns of abundant as well as rare and ephemeral ciliate species determined via HTS correlated with successions observed by morphospecies counting? (b) Does HTS allow for a more precise description of the seasonality of rare and ephemeral ciliate species? (c) Is there a relation between numbers of sequence reads and cellular abundance or biomass values for the ciliate species? (d) Do relative numbers of sequence reads mirror the quantitative assemblage structure determined via morphospecies counting?

## Materials and Methods

### Investigation Site, Sampling and Environmental Parameters

Lake Zurich (47°19.3′N, 8°33.9′E; Switzerland) is a pre-alpine, oligo-mesotrophic, and monomictic lake which is the object of intensive limnological research since five decades (Posch et al., 2012; Yankova et al., 2017). The major morphometric descriptors are: altitude = 406 m a.s.l., maximum depth = 136 m, mean depth = 51 m, area = 65.06 km^2^, volume = 3.3 km^3^, and theoretical water renewal time = 1.2 years. The lake serves as drinking water reservoir for more than 1 million people. Plankton samples were taken biweekly from March 2014 to 2017 (*n* = 74 samples). Lake water was collected from a depth of 5 m with a 5 L Ruttner water sampler. The 5 L water volume of each sample was split in 300 mL for the morphospecies counting method and 4 L for HTS (see below), i.e., the analyzed water came from the same starting volume for both methods (see also the workflow scheme in Supplementary Figure S1). Additionally, net hauls (10 μm mesh size) were collected during the whole sampling period from the upper 20 m to identify, isolate and cultivate living specimens. Accompanying abiotic parameters, i.e., water temperature and oxygen concentration were measured *in situ* for the sampling depth (5 m) with a multiparameter probe (6600 V2, Yellow Springs Instruments, United States). Data for the description of the trophic status, i.e., total phosphorus (TP) and nitrate (NO_3_-N) concentrations, and total abundances of phytoplankton were obtained from the Water Supply Company Zurich. For bacterial counts, 20 mL of each 5 L sample were preserved with formaldehyde (2% final concentration), stained with SYBR Green I (Sigma Aldrich) and evaluated via flow cytometry (Cytoflex S, Beckman Coulter; Petrou and Nielsen, 2018).

### Identification, Abundance and Biomass of Ciliates

Determination of living ciliates from net hauls was done subsequently after sampling in the lab using a Zeiss Axio ImagerM1 microscope (magnification: 100× to 840×) and a Zeiss Discovery.V8 stereo microscope (magnification: 10× to 80×). Single or multiple cells of identified ciliates (Table 1 and Supplementary Figure S1) were isolated using a drawn glass micropipette and washed in 3–5 drops of sterile filtered lake water or medium (Volvic mineral water). Cultures were sustained by sterilized wheat grains supporting bacterial growth for bacterivorous ciliates or by a *Cryptomonas* strain SAG 26.80 (Culture Collection of Algae at University of Göttingen, Germany) serving as food source for algivorous / omnivorous species. Ciliate cultures were kept at 18°C and a 14 h light (20 μmol m^-2^ s^-1^)/8 h dark cycle.

**Table 1:** List of the twelve ciliate species which have been isolated and partly cultivated during the investigation period (March 2014–2017).

Taxonomy	Cultivation successful	Food	Newly sequenced taxa?	Accession number GenBank	Sequence length in bp	Closest known relative in Genbank (similarity; accession number)
Intramacronucleata						
Litostomatea						
*Pelagodileptus trachelioides*	no	–	no	LS999902	757	*Pelagodileptus trachelioides* (100%; AB558117)
Oligohymenophorea						
*Cinetochilum margaritaceum*	yes	wheat grains^c^	**yes**	LR025746	2832	*Urocentrum turbo* (90%; AF255357)
*Histiobalantium bodamicum*	yes	*Cryptomonas* sp.	**yes**	LS999901	1724	*Histiobalantium comosa* (99%; KU665372)
*Stokesia vernalis*	no	–	no	LS999907	300	*Stokesia vernalis* (100%; HM030738)
Prostomatea						
*Balanion planctonicum*^a^	yes	*Cryptomonas* sp.	**yes**	LS999896	826	*Balanion masanensis* (92%; AM412525)
*Coleps* sp. mixotroph	yes	*Cryptomonas* sp.	no	LS999899	1269	*Coleps spetai* (100%; AM292312)
Spirotrichea						
*Codonella cratera*^b^	no	–	no	LS999898	1362	*Tintinnopsis lacustris* (100%; JQ408161)
*Halteria bifurcata*^a^	yes	wheat grains^c^	**yes**	LS999900	1618	*Halteria grandinella* (98%; AF194410)
*Pelagostrombidium mirabile*	yes	*Cryptomonas* sp.	**yes**	LS999903	1676	*Strombidium paracalkinsi* (96%; KJ737432)
*Rimostrombidium lacustris*	yes	*Cryptomonas* sp.	no	LS999904	1582	*Rimostrombidium lacustris* (99%; DQ986131)
*Uroleptus willii*	yes	*Cryptomonas* sp.	no	LS999908	1619	*Uroleptus willii* (100%; EU399543)
Postciliodesmatophora						
Heterotrichea						
*Stentor roeselii*	yes	*Cryptomonas* sp.	no	LS999906	1289	*Stentor roeselii* (100%; KP970248)


*(a) *Incertae sedis*, Liu et al. (2015) and Gao et al. (2016), respectively. (*b*) Synonym of *Tintinnopsis lacustris*, Bachy et al. (2012). (*c*) Mixed bacterial suspension growing on wheat grains. Specific sequences of the V9 regions (18S rRNA gene) were determined for all listed ciliates. Five of these species were not represented in databases up to now. We intended to get as complete sequence information (up to 2832 bp) about the rRNA genes as possible.*

For counting and biomass calculations, 300 mL of lake water were preserved with freshly prepared 15 mL Bouin’s solution [containing 10.7 mL saturated picric acid, 3.6 mL formaldehyde (37% stock solution) and 0.7 mL glacial acetic acid; Skibbe, 1994]. Subsamples (100–150 mL) were filtered through 0.8 μm cellulose nitrate filters equipped with counting grids (Sartorius, Germany). Then, the quantitative protargol (silver proteinate) staining (QPS) method was applied following the protocol of Skibbe (1994) with slight modifications after Pfister et al. (1999). After the staining procedure, silver impregnated ciliates on filters were embedded in Canada balsam (Merck, Germany) providing permanent slides which keep their quality for years. Preparations were analyzed at 200× to 1,600× magnification with a Zeiss Axio ImagerM1 microscope. The identification of living and stained ciliate species was done according to Foissner et al. (1991, 1992, 1994, 1995, 1999) and Sonntag et al. (2008). We followed the classification systems by Lynn (2008) and Gao et al. (2016) for taxonomic affiliation of detected species. For each sample we inspected an equivalent filter area until at least 400 cells were determined and counted. Finally, the total filter area was checked for rare species which would have been overlooked by our routine counting procedure. Biomass (i.e., fresh weight) for each identified ciliate species was calculated by multiplying ciliate abundances with species-specific conversion factors published by Foissner et al. (1992, 1994, 1999). These published factors are based on average cell size of a species, a geometric approximation of the cell form to calculate cell volume and a specific density of 1 pg μm^-3^ (for details see chapter 2 in Foissner et al., 1992).

### Sequencing of Isolated Ciliate Species

Partial 18S rRNA genes including the V9 region were sequenced either from cultivated ciliates or directly from living individuals gathered from net hauls (Table 1 and Supplementary Figure S1). Cells were washed as described above and starved for ∼24 h in sterile filtrated lake water or medium to ensure that all potential food particles were digested. Subsequently, ciliates were transferred to 1–2 μL PCR grade water which was then directly used as template for a nested PCR using the primers from Marin et al. (2003; see Supplementary Figure S2). Alternatively, the primers Euk360F (Edgcomb et al., 2011) and Univ1492RE (Stoeck et al., 2006) were used for our standard PCR protocol (Supplementary Figure S2).

Conditions for the nested PCR were as follows: First denaturation at 96°C for 1 min followed by 30 cycles, each consisting of 1 min at 96°C, 2 min at 55°C, 3 min at 72°C, followed by a final elongation of 10 min at 72°C using the GoTaq^®^ Green Master Mix (Promega). This procedure was successfully applied for the species *Cinetochilum margaritaceum* (Table 1). The partial 18S rDNA of the remaining eleven species was amplified using the standard PCR protocol. The conditions were as follows: First denaturation at 94°C for 3 min followed by 35 cycles, each consisting of 1 min at 94°C, 1 min at 52°C, and 2 min at 72°C, followed by a final elongation of 5 min at 72°C using the GoTaq^®^ Green Master Mix (Promega). PCR products were purified with Agencourt AMPure XP PCR Purification Kit (Beckman Coulter) and Sanger sequenced with ABI BigDye chemistry on an ABI 3130x Genetic Analyzer (Applied Biosystems). In some cases, additional sequencing primers E528F (Marin et al., 2003) and SR10f (Nakayama et al., 1998) were used in order to sequence the entire V9 region.

### DNA Extraction, Amplification and Sequencing

Raw water samples (2 × 2 L) were pre-filtered through a 150 μm net to remove larger zooplankton and subsequently, filtered onto a 0.65 μm membrane filter (Durapore, Millipore) using a peristaltic pump with a low flowrate of 50 mL min^-1^. The filters (duplicates) were directly transferred to a cryovial containing 1.5 mL RNAlater (QIAGEN), placed in the refrigerator (∼5°C) overnight and finally stored at -80°C until further processing. For DNA extraction, each filter was transferred into a lysing matrix tube (Lysing Matrix E, MP Biomedicals) and 600 μL RLT buffer and 6 μL β-Mercaptoethanol were added. The remaining liquid in each cryovial was centrifuged and discarded. To the residual pellet, 200 μL RLT buffer and 2 μL β-Mercaptoethanol were added and mixed. This mix was added to the lysing matrix tube. To break and remove cells from the filter, each matrix tube was subjected to bead-beating for 45 s by 30 Hz. Total environmental DNA was then extracted using AllPrep DNA/RNA Mini Kit (QIAGEN).

The hypervariable V9 region (about 150 bp long) of the 18S rDNA was amplified from the extracted DNA following the protocol of Stoeck et al. (2010). The forward primer was 1391F (5′-GTACACACCGCCCGTC-3′; referring to position 1629–1644 in the *Saccharomyces cerevisiae* reference with accession number U53879 in NCBI’s GenBank; Lane, 1991) and the reverse primer EukB (5′-TGATCCTTCTGCAGGTTCACCTAC-3′; referring to position 1774–1797 in *S. cerevisiae*; Medlin et al., 1988). The PCR protocol for V9 amplification employed an initial denaturation step at 98°C for 30 s, followed by 30 cycles of 10 s at 98°C, 20 s at 61°C, 25 s at 72°C and a final 5-min extension at 72°C. The reactions were run in volumes of 50 μL using 0.5 μL Phusion polymerase (Biolabs), 10 μL 5× Phusion GC buffer (Biolabs), 1 μL 10 mM dNTPs, 0.5 μL template DNA, 32.5 μL pure water, and 0.5 μL forward, and 0.5 μL reverse primers. Successful amplification was checked with agarose gel electrophoresis using 1.0 g agarose (Carl Roth GmbH), 100 mL TAE buffer (1×) and 5 μL ethidium bromide. From each DNA extract, triplicate PCR reactions were conducted to minimize PCR bias. Prior to purification (Qiagen’s MinElute Kit), PCR replicates obtained from the same DNA extract were combined.

From the resulting PCR products, sequencing libraries were constructed using the NEB Next^®^ Ultra^TM^ DNA Library Prep Kit for Illumina (NEB). The quality of the libraries was assessed with an Agilent Bioanalyzer 2100 system. Eighteen samples of the time-series dataset (March 2014 – October 2014) were sequenced on an Illumina NextSeq platform, generating 250-bp-paired-end reads. All other samples (November 2014 – March 2017) were sequenced on an Illumina MiSeq platform, generating 300-bp paired-end reads. High-throughput sequencing was conducted by SeqIT GmbH & Co. KG (Kaiserslautern, Germany). We used at least 250 bp-NextSeq reads to guarantee the highest possible sequence quality (100% overlap of read 1 and read 2 sequences; please note: sequences were exceeding 150 bp in length due to multiplexing and adapters).

### Pre- and Postprocessing of High Throughput Sequencing Data

Paired-end reads were merged using a custom script. The quality of the HTS data was evaluated as following: In an initial step, excessive primer overhangs were clipped with CUTADAPT version 1.18 (Martin, 2011). Reads were then quality-filtered using the *split.libraries.py* command in QIIME version 1.8.0 (Caporaso et al., 2010). Only such reads were retained which had exactly matching barcodes and primers, contained exclusively unambiguous nucleotides and had a minimum length of 90 basepairs. In the final quality-filtering step, all reads underwent *de novo* chimera analysis in UCHIME v5.2.236 (Edgar et al., 2011).

All high-quality reads were eventually de-replicated into amplicons and clustered in SWARM version 2.2.2 (Mahé et al., 2015) using *d* = 1. This value *d* refers to a local clustering threshold instead of an arbitrary global clustering threshold. Additionally, SWARM does not depend on the input order. OTU grow iteratively by comparing each generation of assigned amplicons to the remaining reads in the dataset. An OTU is closed when no new amplicon with *d* or fewer differences can be assigned to the OTU. Using a custom script, an OTU contingency table was created based on the output files of SWARM (the script can be directly obtained upon request from Dominik Forster, University of Kaiserslautern).

### Assignment of HTS Data to Cultured Ciliate Species

Prior to comparing HTS data to sequence data of cultured ciliate species, the OTU contingency table was normalized to the smallest number of sequences in all samples (110,100 sequences in sample March 31, 2014). Normalization was done in R version 3.5.1 (R Development Core Team, 2008) using the *rrarefy* command in the package *vegan*^1^. From each OTU, we extracted the seed sequence as a representative. The representatives of the HTS dataset were then compared against the sequences obtained from the twelve selected ciliate species using *blastn* in BLAST version 2.6.0 (Altschul et al., 1990). HTS sequences had to share a fragment of at least 90 consecutive nucleotides and a similarity of 97% in order to be assigned to one of these twelve cultured ciliates. Only those OTUs whose representative sequences matched our twelve selected ciliates (Table 1) were extracted from the normalized contingency table for further analyses.

### Statistical Analyses: Comparison of Molecular and Morphological Data

To test if succession patterns of morphospecies counts were resembled by HTS data, a Spearman’s rank-order correlation coefficient was calculated for each of the twelve investigated ciliate species. As we performed several correlation tests on the same data set, a Bonferroni correction was applied to protect from an inflation of the alpha level. In addition, cell counts, biomass values and numbers of sequence reads per sampling date were used to compile three dissimilarity matrices (Bray Curtis) – one for each measurement. Correlation of sequence versus cell counts or biomass matrices was tested using a simple Mantel test with 10,000 permutations. All statistical analyses were performed using R version 3.4.4 (R Development Core Team, 2008).

## Results

### Seasonality of the Ciliate Assemblage With Special Focus on Twelve Selected Species

**FIGURE 1:**
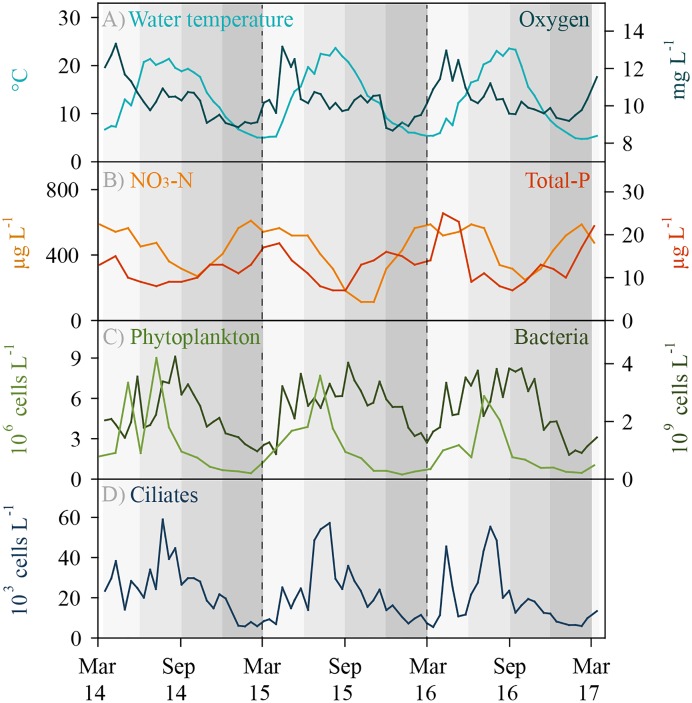
Environmental data and total ciliate abundance for Lake Zurich determined for the 5 m depth layer from March 2014 to 2017 (biweekly sampling, *n* = 74). **(A)** Water temperature (°C) and oxygen concentration (mg L^-1^). **(B)** Total phosphorus (μg L^-1^) and nitrate NO_3_-N (μg L^-1^) concentrations. **(C)** Total phytoplankton abundance (10^6^ cells L^-1^) and total heterotrophic bacterial abundance (10^9^ bacteria L^-1^). **(D)** Total ciliate abundance (10^3^ ciliates L^-1^) determined by morphospecies counting of protargol impregnated specimens.

Lake Zurich is monomictic and water turnover usually occurs in early springtime, i.e., between February and April. Environmental parameters showed typical seasonal patterns, i.e., there were no extreme climatic years during the investigation period (Figure 1). Water temperature in the sampling depth (5 m) reached a minimum of 4.7°C in winter and a maximum of 23.7°C in summer. Maximal oxygen concentrations were measured during the phytoplankton spring bloom phases (April–May). Toward the end of the year, oxygen concentrations slightly declined, however, values in 5 m depth were still above 8.7 mg L^-1^ (>70% saturation) even during winter. Total phosphorus and nitrate (NO_3_-N) concentrations decreased owing to uptake by autotrophs toward the end of the year and increased as a result of water turnover in early spring. Maximal abundances of phytoplankton were reached during summer months, whereas numbers of total heterotrophic bacteria increased during spring and remained at a high level (ca. 4 × 10^9^ bacteria L^-1^) until autumn. Total ciliate abundances followed typical repetitive annual patterns with slightly pronounced peaks during springtime and maximal values (ca. 60 × 10^3^ cells L^-1^) during summer at highest water temperatures (Figure 1). During the 3 years of investigation, we detected 48 different ciliate morphotypes based on microscopic counts, and 31 of these morphotypes could be clearly assigned to morphospecies level (Supplementary Table S1). Seventeen morphotypes were assigned only to genus level, partly (i) representing yet undescribed species and (ii) morphotypes which could not be linked to a known species owing to methodological restrictions in the evaluation of QPS preparations.

**FIGURE 2:**
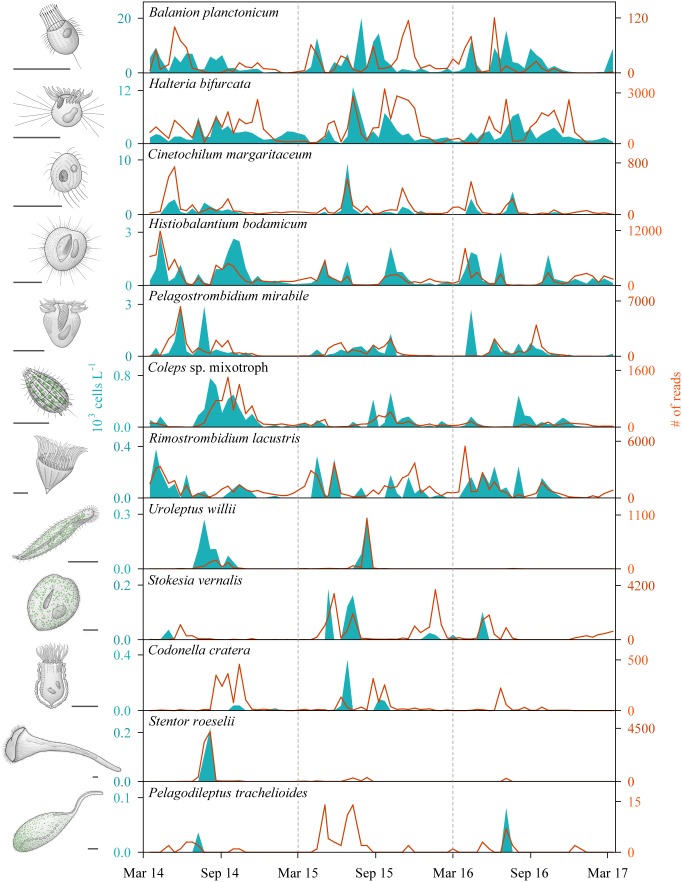
Seasonal successions of twelve selected ciliate species determined by morphospecies counting of silver impregnated specimens (areas) and high throughput sequencing (HTS) of the V9 regions of the 18S rRNA gene (lines) during the 3 years of investigation (*n* = 74 for each species and applied method; sampling depth = 5 m). The order of species reflects their average abundance based on counting, with the most abundant species on top and the rarest representative on bottom. Sketches of ciliate morphospecies are original drawings from Gianna Pitsch. Scale bars: 40 μm.

**FIGURE 3:**
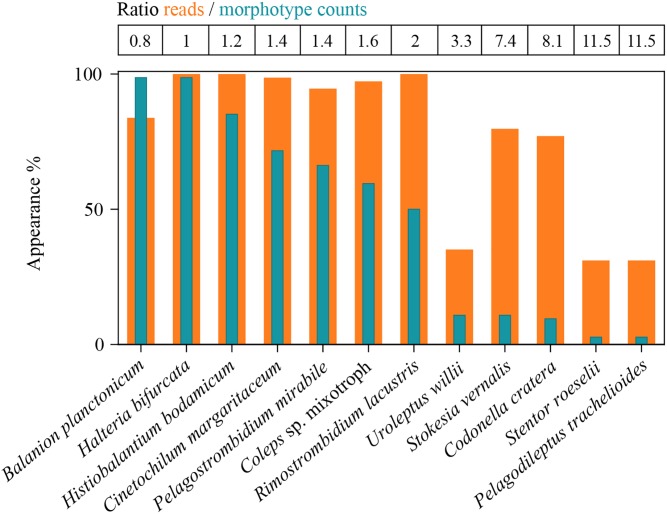
Detection of the twelve selected species in all analyzed samples (100% = 74 samples) by morphotype counting and HTS. Ratios of reads- versus counts-appearances are listed as table above the panel. Due to method specific restrictions, the total inspected sample volume was in maximum 150 mL for the microscopic approach. HTS data are based on 4 L of filtered sample volume.

With our isolation and cultivation workflow (Supplementary Figure S1), we determined specific sequences (including V9 regions of the 18S rRNA gene) for twelve ciliate species assigned to five classes from Lake Zurich that were used as seeds for HTS analyses (Table 1, accession numbers: LR025746, LS999896, LS999898-LS999904, LS999906-LS999908). Five of these species were not yet represented in public genetic databases. Several highly abundant and very frequent species could be characterized by morphospecies counting as well as HTS (Figure 2). The comparison of both methodological approaches resulted in the following outcomes: (i) Seasonal successions for the most abundant morphospecies based on counts showed remarkable correlations with HTS data (see seven top panels in Figure 2, Table 2, and Supplementary Figure S3). Even for species with striking abundance fluctuations, e.g., *Cinetochilum margaritaceum, Histiobalantium bodamicum*, *Pelagostrombidium mirabile*, a mixotrophic *Coleps* sp., and *Rimostrombidium lacustris* both methods gave highly comparable temporal dynamics. However, the correlation of reads specific for *Balanion planctonicum* with cells counts was rather low (see discussion for possible reasons). A moderate correlation between the two methods was observed for the second most abundant species *Halteria bifurcata*. *Halteria* counts probably comprised two barely distinguishable species, namely *H. bifurcata* and *H. grandinella*, leading to potential errors in morphospecies counts. Nevertheless, there were statistically significant positive correlations (Spearman’s rank-order) between microscopic counts and HTS data for eleven of the twelve selected ciliates, but high coefficients >0.6 were determined for only five species (Table 2). For eight species, significant correlations were found for the whole 3 years’ dataset, but not within each year (Table 2). (ii) Divergence between HTS and morphospecies counting was noticed for ephemeral and rare species, based on morphological investigations (e.g., *Uroleptus willii*, *Stokesia vernalis*). Here, HTS seemed to be a more precise method to describe their seasonality than the classical approach (Figure 2, 3 and Supplementary Figure S3). For example, HTS demonstrated the annual appearance for *Codonella cratera* and *Pelagodileptus trachelioides* in all 3 years of the study, although these species could be found only in 2 of 3 years by microscopy. The largest discrepancy between both methods was observed for *Stentor roeselii*. We detected this species microscopically only in 1 year (i.e., in one out of 74 analyzed protargol impregnated slides), whereas amplicon reads demonstrated the appearance in all 3 years of investigation (Figure 2).

**Table 2:** Spearman rank order correlation analysis of abundances for the twelve selected ciliate species versus the number of reads for their specific V9 sequences.

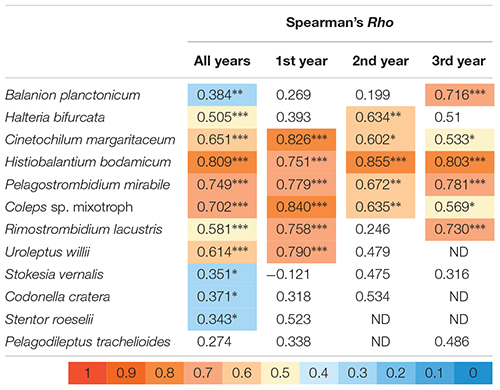

*Values were determined for all 74 samples from March 2014 to 2017, and for each year of investigation separately. Levels of significance: ^∗∗∗^(*p* < 0.001), ^∗∗^(0.001 < *p* < 0.01), ^∗^(0.01 < *p* < 0.05). *P*-values denote Bonferroni-adjusted significance. Color codes indicate significant correlations and the height of Spearman’s Rho coefficient. ND = not determined.*

In sum, HTS showed higher percentages of occurrence for all except of one ciliate species (*B. planctonicum*) than microscopic counts (Figure 3). Differences between the two methods were striking for some ciliates, e.g., specific reads for *R. lacustris* were recorded for all investigated samples (*n* = 74), but we found this species only in 50% of inspected QPS preparations. Based on cell counts, we would have defined *S. vernalis* and *C. cratera* as truly ephemeral species, however, HTS data showed their appearance in 80 and 77% of samples, respectively (Figure 3).

### Abundance and Biomass Values Versus Numbers of Sequence Reads

**FIGURE 4:**
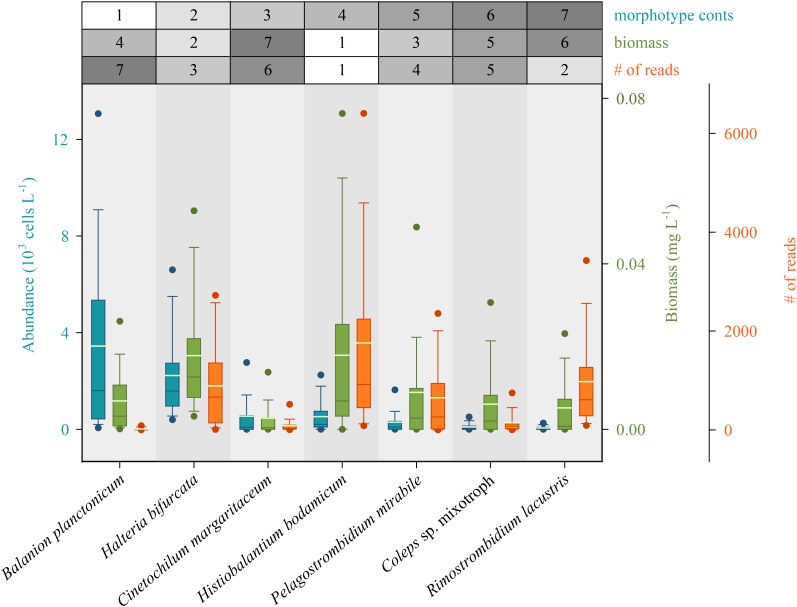
Rank order of the seven most abundant ciliate species based on abundances compared to their biomasses and the number of species specific reads (HTS) during the 3 years of investigation (*n* = 74 for each species). Method specific rank orders are listed in the table above the panel. Bars show the 25th, 50th, and 75th percentiles, lines in each bar show medians, whiskers stand for the 10th and the 90th percentiles and points indicate the 5th and 95th percentiles. The white lines in bars are averages.

We first defined rank orders for the seven most abundant species based on numbers, and determined if these classifications were also reflected by biomasses and numbers of species-specific sequence reads (Figure 4). Congruence between number of sequence reads and biomasses was notably higher than between number of sequence reads and morphospecies abundances. However, for some species we found striking under- or overestimations of their quantitative importance in the ciliate assemblage by HTS. For example, counts identified the small *B. planctonicum* (∼20 × 15 μm) as the numerically dominant species throughout the investigation period, while numbers of sequence reads for this specific taxon were low compared to all other studied ciliates (Figure 4 and Supplementary Table S2). An opposite ratio was detected, e.g., for the larger species *R. lacustris* (120 × 70 μm), which was characterized by high numbers of sequence reads but low abundances of counted individuals (Figure 2, 4 and Supplementary Table S2). Throughout the study, highest numbers of sequence reads were obtained for *H. bodamicum*, a species which reached also high total biomass values (Figure 4).

**FIGURE 5:**
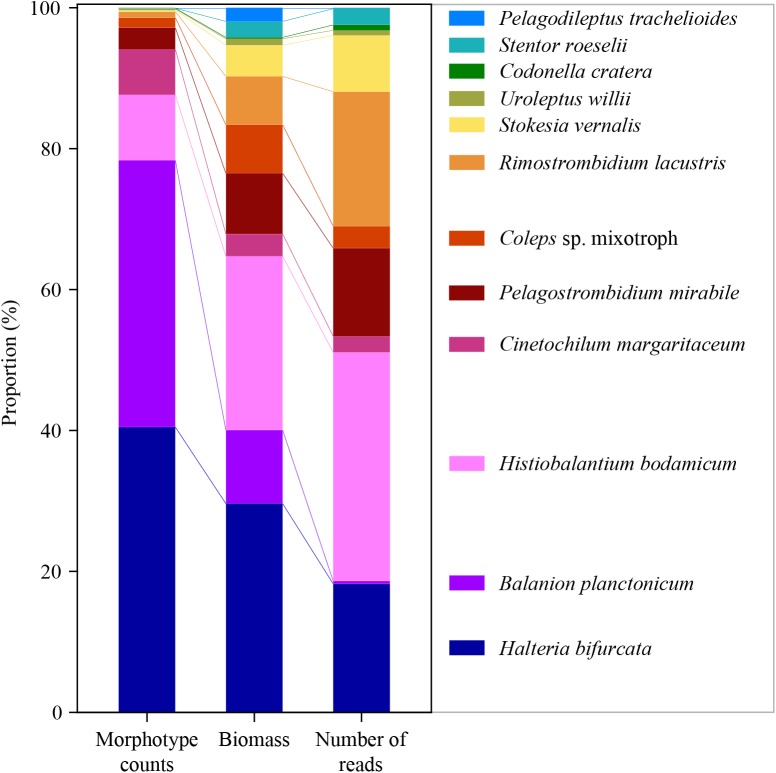
Ratios of the twelve selected species to each other in relation to abundance (left), biomass (middle) and number of reads (right) for the whole period of investigation (average of 74 samples for each species and applied method).

For a final overview, we compared ratios of the twelve selected species to each other in relation to abundance, biomass and number of sequence reads for the whole period of investigation (Figure 5). Due to differences in the average cell sizes of these species, the contribution to biomass differed from their proportion of cellular abundances. For example, *B. planctonicum* contributed with 38% to the cumulative abundance of the twelve selected species, but accounted for only 11% of cumulative biomass and for 0.5% of sequence reads, respectively. An opposite pattern was observed for, e.g., *R. lacustris* with contributions of 0.9, 7, and 19% to cumulative abundance, biomass and reads values, respectively. In summary, ratios of species specific reads to each other better reflected the assemblage composition based on biomass determination (Figure 5). This trend was supported when comparing Bray Curtis distances between each pair of samples (*n* = 74) using cell counts, biomass and the number of sequence reads for each species. A Mantel test indicated a stronger correlation between distances based on the number of sequence reads and biomass per species (*r* = 0.425, *p* < 0.001) rather than that based on the number of sequence reads and cell counts per species (*r* = 0.231, *p* < 0.001).

## Discussion

### Strengths and Limitations of Morphospecies Based Quantifications

Since the concept of the microbial loop and its importance for food web dynamics was first formalized (Azam et al., 1983), research on the role of ciliates as intrinsic components of marine and freshwater pelagic systems has received considerable attention. Today, we can profit from a “long” lasting research history in ciliate morphospecies analyses – with a special focus on plankton. Since the 1990s, new methodological approaches (Montagnes and Lynn, 1987; Skibbe, 1994) and excellent taxonomic compilations (Foissner and Berger, 1996; Foissner et al., 1999) allowed for detailed descriptions of ciliate assemblage successions. Thus, seasonal patterns of some key planktonic species are well described, including data on environmental factors (e.g., food sources, temperature effects, top-down grazing pressure) which cause ciliates’ successions (see Foissner et al., 1999; and references therein). Consequently, for any study on planktonic ciliates, a correct morphospecies identification is the prerequisite to make use of this profound knowledge. Here the quantitative protargol staining (QPS) proved as the tool of choice to quantify ciliates in combination with a relatively high taxonomic resolution (Montagnes and Lynn, 1987; Jerome et al., 1993; Skibbe, 1994).

Due to our long work experience with this method (Pfister et al., 1999), we can define its pros as well as its drawbacks and limitations: (i) As preserved and stained specimens are concentrated on filters and embedded in a layer of agar first and in Canada balsam in the end, preparations have a certain thickness which may negatively affect the optical evaluation during bright-field microscopy. This is especially problematic for the identification of very tiny species, where minute differences, e.g., in their oral or somatic ciliary patterns, need to be recognized. For example, it is difficult to discriminate between small morphospecies within the genera *Urotricha* and *Cyclidium* in QPS slides (Supplementary Table S1). Thus, non-quantitative silver impregnation methods are needed additionally for an exact species identification (Foissner, 2014). (ii) The entire work procedure of QPS is a time-consuming method and commonly 5 h are needed to stain 8 samples, and usually several hours have to be spend for the evaluation of one preparation. As a consequence, we have restrictions in sampling campaigns which aim in high spatial and temporal resolutions of ciliate assemblage dynamics. (iii) All methods for the quantification of planktonic ciliates only allow for the analysis of a limited water volume. For example, we could concentrate a maximum of 150 mL per sample to obtain good preparations for the oligo-mesotrophic Lake Zurich.

The two latter drawbacks (restrictions of evaluable samples and water volumes) seem to hamper the analysis of ephemeral and rare species on the one hand, but also the description of population dynamics of abundant and fast growing representatives on the other hand. In an earlier study on the spring bloom dynamics in Lake Zurich, we found by high sampling frequency (2–4 days sampling intervals) that vernal abundance peaks of numerous species lasted for only a few days (Posch et al., 2015). Such rapid population dynamics were observed in several lakes (e.g., Müller et al., 1991; Šimek et al., 2014) and were linked to high growth rates of ciliates resulting in doubling times of 1–2 days (Müller and Geller, 1993; Macek et al., 1996). Consequently, there is definitely need to investigate larger sample volumes at a high temporal resolution (e.g., daily during the warm season) to elucidate dynamics of planktonic ciliate assemblages. Here, HTS seems to be a promising supplementary approach to morphospecies-based methods.

### The Choice of the V9 Region as Marker

Different hypervariable regions have been used for the monitoring of eukaryotic plankton communities in previous studies, and their utility has been debated among researchers. For a review and a comparison, we refer to Tanabe et al. (2016). Among the three most popular marker regions (V1–V2, V4, and V9), we chose the V9 region for the following reasons: (i) While primer universality is highly similar for all three specific standard primer sets, currently, the V9 region is the best tradeoff between database coverage and taxonomic resolution (sequence variability; Tanabe et al., 2016). (ii) Furthermore, this region is notably shorter than the V1–V2 and V4 regions and thus, allows for a much larger sequence depth at lower costs, which is especially relevant in studies with high sample numbers. (iii) Finally, the largest environmental sequencing study for eukaryotic plankton communities available to date (De Vargas et al., 2015) used the V9 region, allowing for a good coverage of most (if not all) major eukaryotic taxonomic lineages. Future studies on environmental sequence metadata from eukaryotic plankton will benefit from surveys, which already used this genetic marker.

### Correlations of HTS Data With Morphotype Counting

Translating amplicon abundance of HTS datasets into cell abundance is a critical issue in molecular ecology and environmental sequencing. In earlier studies of protistan mock communities, while all species were detected after HTS sequencing, the relative proportion of sequence types did not correlate with the cell abundances in the cell mixtures (Egge et al., 2013; Weber and Pawlowski, 2013). In addition, comparative studies in natural ecosystems showed that sequence abundances and cell abundances are often incongruent (Bachy et al., 2013; Santoferrara et al., 2014; Stoeck et al., 2014). However, these studies are not conclusive, because other studies support the quantitative use of HTS data. Giner et al. (2016) reported adequate correlations between microscopy counts of planktonic picoeukaryotes and molecular signals after HTS. This is supported by more recent studies of Forster et al. (2018) and Stoeck et al. (2018), showing that assemblage analyses based on ciliate HTS abundance data mirrors patterns obtained from morphological investigations of the same samples. Giner et al. (2016) argued that PCR biases (Stoeck et al., 2006; Shakya et al., 2013; Tragin et al., 2018) and putative sequencing artifacts (Huse et al., 2007) do not affect the correlation between relative read and cell abundances. The authors demonstrated that more reads imply a higher proportion of cells. According to these authors, the use of relative read abundance as a proxy of community composition for comparative purposes is justified. The contradicting results of the mentioned studies demonstrate that relative abundances of HTS data may be interpreted quantitatively, but also warn about considerations as direct proxies for cell abundances in natural communities. The circumstances under which quantitative analyses and interpretations of HTS data are possible and reliable are still to be defined.

In our study, both methods showed clear seasonal successions for all selected species and HTS based population dynamics were in most cases remarkably correlated with morphospecies data (Figure 2 and Supplementary Figure S3). Up to now, there were only few attempts to compare temporal HTS datasets with microscopic analyses for freshwater systems. Medinger et al. (2010) found significant correlations between cell abundance and sequence data only for two out of five investigated protistan species. Stoeck et al. (2014) conducted a survey of the ciliate assemblage from an oligo-mesotrophic lake by microscopy and pyrosequencing of the 18S rRNA gene V4 region. In the latter study, sequencing data did not mirror morphospecies abundances at all. One major reason for this discrepancy was an inadequacy of the reference gene database with extremely low coverage of typical freshwater ciliates. The authors already stated that further barcoding initiatives for freshwater ciliates (not only focused on the V4 region) were urgently needed to fully exploit the potential of HTS in ecological studies of freshwater protists. Also, a deeper sequencing strategy as achievable with pyrosequencing would be beneficial to cover the full extent of protistan plankton diversity in freshwater environments (Stoeck et al., 2014).

As shown by our study, ciliates’ succession dynamics based on read abundances were very similar to patterns deduced from morphospecies counts. Two steps toward a better congruence of patterns obtained from microscopy and sequencing are (i) a deeper sequence coverage through Illumina sequencing technology and (ii) the availability of ciliate reference sequences obtained from the indigenous assemblage members. By this workflow, HTS helped indeed to shed light on population dynamics of ephemeral and rare species (Figure 2). The advantage of HTS over microscopy in this context was certainly the possibility to screen higher samples volumes (HTS: 4 L, morphospecies analysis: max. 150 mL), which is a prerequisite to address this question (Dolan and Stoeck, 2011). Since rare species may fall below the limit of microscopic detection during certain periods of the year, amplicon reads will still be found by HTS. Conversely, sequencing does not allow to distinguish between cells appearing as active trophozoites or as resting cysts in the pelagial. Thus, population dynamics of ephemeral and rare ciliate species are still a topic for further research.

*Balanion planctonicum* represented the only species, which was detected in more samples by microscopy than by HTS (Figure 2, 3). For the interpretation of HTS data, we could make use of only one reference sequence obtained from a clonal *B. planctonicum* strain (Table 1). However, this morphospecies may represent a species complex harboring cryptic taxa, which would not have been detected by our workflow, and sequencing of more clonal cultures will be needed in future. The following observation supports our thesis: By setting an identity threshold of 99% in comparison to the applied 97%, we received highly similar numbers of specific reads for all species except for *B. planctonicum*, which could not be detected using the higher threshold level (Supplementary Table S2). In addition, the largest discrepancy between morphological counts and amplicon read abundances was observed during summer months, when total ciliate abundances reached maximal values. As numbers of *B. planctonicum* specific reads were *per se* marginal, amplicon signals probably got lost in relation to high numbers of all other ciliate reads owing to normalization routines of HTS analysis.

### Relationship Between Numbers of Reads and Abundance or Biomass Values

We examined if the ratios of reads between the twelve chosen species mirrored the assemblage composition deduced from cell counts (Figure 5). Divergence between the two methods could be expected (Santoferrara et al., 2014; Stoeck et al., 2014), as probably all ciliate species have an extremely high copy number of the rDNA operon per individual cell. For example, Gong et al. (2013) reported a value of ∼316,000 (±7,100 standard deviation) copies per cell for a peritrichous ciliate (*Vorticella* sp.). The authors also documented, that rDNA copy numbers per cell markedly differed among (from 3,385 to 315,786) and even within 14 investigated isolates. The latter variation may be linked to macronuclear formation or disintegration during different growth and sexual phases of the cell. For our study, species specific variations in rDNA operon copy numbers may be the reason why some, even abundant, species (e.g., *B. planctonicum* and *C. margaritaceum*) were characterized by low numbers of reads. In contrast, reads of *H. bodamicum* reached highest values throughout the study, although being not the most abundant species. Systematic under- or over-representations of species specific reads could also be induced by primer specific amplification patterns. However, the relative high congruence of assemblage compositions based on biomasses and numbers of reads (Figure 5) rather speaks for a relation between species cell size and rDNA copy numbers. Such a relation was previously shown for 18 algal strains representing several eukaryotic classes (Zhu et al., 2005). Fu and Gong (2017) published the only study investigating the linkage between rDNA and rRNA copy numbers and phenotypic traits in ciliates. The relationship between rDNA or rRNA and cell size (volume) of studied ciliates (ribotype scaling) was in agreement with both the metabolic theory of ecology and the growth rate hypothesis, giving a quantitative framework to link cellular rRNA copy numbers with cell size, growth (activity), and biomass stoichiometry. These results may explain the observed congruence between taxonomic marker gene abundances and cellular biomass in natural ciliate assemblages.

### The Need for Metabarcoding Planktonic Freshwater Ciliates

In this methodological study, we did not focus on the obvious strengths of HTS for analyzing planktonic ciliates, i.e., (i) to elucidate the beta-diversity during the seasonal cycle, (ii) to get indications of cryptic, closely related species, and (iii) to discover probably yet unknown and undescribed species (Santoferrara et al., 2016). It was our aim to compare HTS data with morphotype counts, in respect to the seasonality of abundant as well as rare species. We were aware that sequence information (18S rDNA) for most of the known morphospecies in Lake Zurich (Posch et al., 2015) did not exist at the start of our study. Our selection of the twelve species which were studied in more detail, partly depended on successful isolation and cultivation of specimens. Based on our morphospecies counts, we found that several species within the genus *Urotricha* (Foissner and Pfister, 1997) were as abundant as *B. planctonicum* during the 3 years of investigation (Supplementary Table S1). However, we did not succeed in the isolation and sequencing of *Urotricha* spp., and unfortunately there is not a single entry in public sequence database concerning the V9 region of the genus. Thus, we could not extract *Urotricha* specific reads from our HTS data, illustrating the need for further DNA barcoding of morphologically well described planktonic freshwater ciliates. Based on a comprehensive literature survey, Foissner et al. (1999) assumed that around 180 described morphospecies may be indeed euplanktonic, and that the total number of planktonic species in one lake (alpha diversity) may vary between 50 and 100 (see also Sonntag et al., 2006; Zingel and Nõges, 2010). For Lake Zurich, we detected 48 different ciliate morphotypes based on microscopic counts for the epipelagic realm during the 3 years of investigation. This number seems to be rather low in comparison to the expected and partly described diversity in soils or running waters (see Table 3.7 in Foissner et al., 1999). However, ciliates in pelagic realms need special adaptations on planktonic life and unique strategies to encounter sufficient food particles in often nutrient-poor environments. Taking these numbers as approximation of planktonic ciliate diversity, initiatives for DNA barcoding are manageable even when cryptic or still undescribed taxa may be expected. However, it remains challenging, as many ciliate species show very pronounced seasonal changes with distinct population maxima lasting for a few days or weeks only and being rare for most of the year.

Finally, the evaluation of HTS depends on a substantial database of reference sequences. Through linking OTUs with known morphospecies, we can use the vast knowledge about the autecology of these species. This aspect is crucial for science and especially for applied studies that are based on the identification of indicator species.

## Author Contributions

TP, TS, BS, and GP conceived and designed the study. GP and EB did the morphospecies counts and the sequencing of isolated/cultivated species. TS, ZQ, and DF conducted and evaluated the HTS analyses. All authors discussed the results and contributed to the manuscript concept. GP and TP wrote the manuscript and created all figures and tables.

## Conflict of Interest Statement

The authors declare that the research was conducted in the absence of any commercial or financial relationships that could be construed as a potential conflict of interest.

## Footnotes

1 https://cran.ism.ac.jp/web/packages/vegan/vegan.pdf
