# Supplementary material for: Seasonality of Planktonic Freshwater Ciliates: Are Analyses Based on V9 Regions of the 18S rRNA Gene Correlated With Morphospecies Counts?
Source: Front Microbiol. 2019 Feb 19;10:248. doi: 10.3389/fmicb.2019.00248 (PMC6389714; doi:10.3389/fmicb.2019.00248)
Supplement: Supplementary file 1 [file Data_Sheet_1.pdf]

Seasonality of planktonic freshwater ciliates: Are analyses based on V9 regions of the 18S rRNA gene correlated with morphospecies counts?

Gianna Pitsch[1], Estelle Patricia Bruni[1], Dominik Forster[2], Zhishuai Qu[2], Bettina Sonntag[3], Thorsten Stoeck[2], and Thomas Posch[1]*

[1] Limnological Station, Department of Plant and Microbial Biology, University of Zurich, Kilchberg, Switzerland
[2] Ecology Group, University of Technology Kaiserslautern, Kaiserslautern, Germany
[3] Research Department for Limnology - Mondsee, University of Innsbruck, Mondsee, Austria

Supplementary Information

Supplementary Figures & Legends S1 – S3 and Table S1 - S2

* Corresponding author:
Thomas Posch, University of Zurich, Department of Plant and Microbial Biology, Limnological Station, Seestrasse 187, CH-8802 Kilchberg, Switzerland
Phone: +41 44 63 49224, fax: +41 44 63 49225, email: posch@limnol.uzh.ch

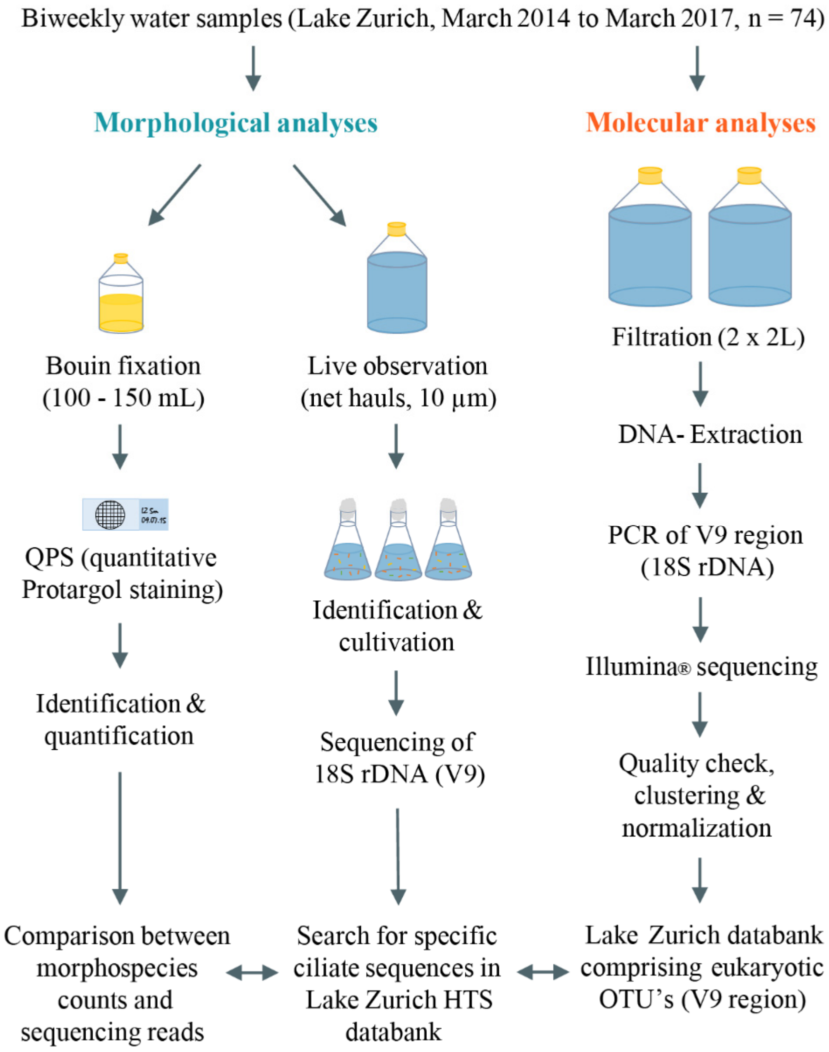

Supplementary Figure S1 Scheme of the workflow to characterize the seasonality of planktonic freshwater ciliates based on morphospecies counts and high throughput sequencing (HTS). In addition, we isolated, cultivated and sequenced selected species to obtain new sequence information for representatives which were not represented in public databases up to now.

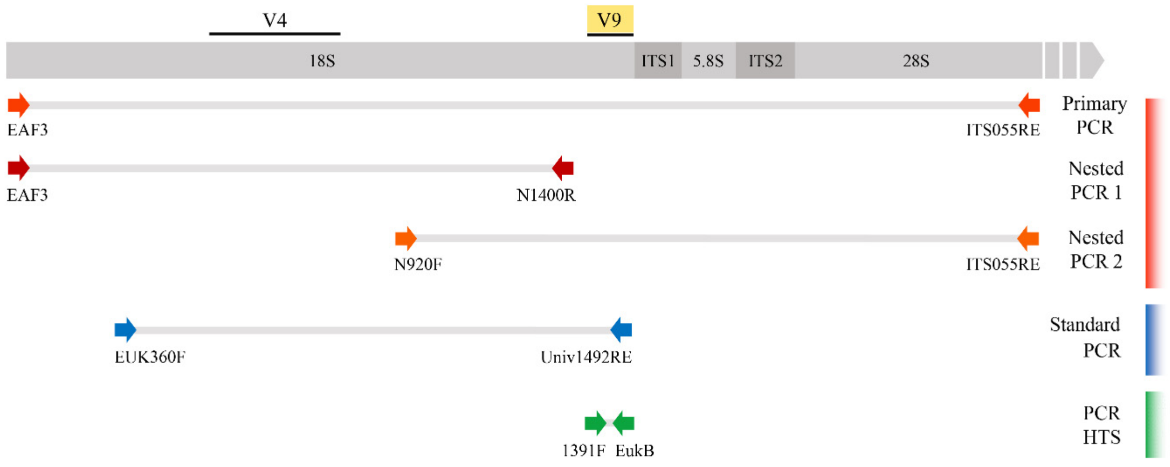

Supplementary Figure S2 Graphical overview of the applied primer sets (arrows) to gain rRNA genes sequences for the isolated and cultivated ciliate species in this study. In addition the primer pair used for the high throughput sequencing (HTS) approach is presented. A major focus was set on the V9 region, however, it was our intention to get as complete sequence information about the rRNA genes as possible. Arrows are not scaled to the real lengths of primers. For further details see Material and Methods.

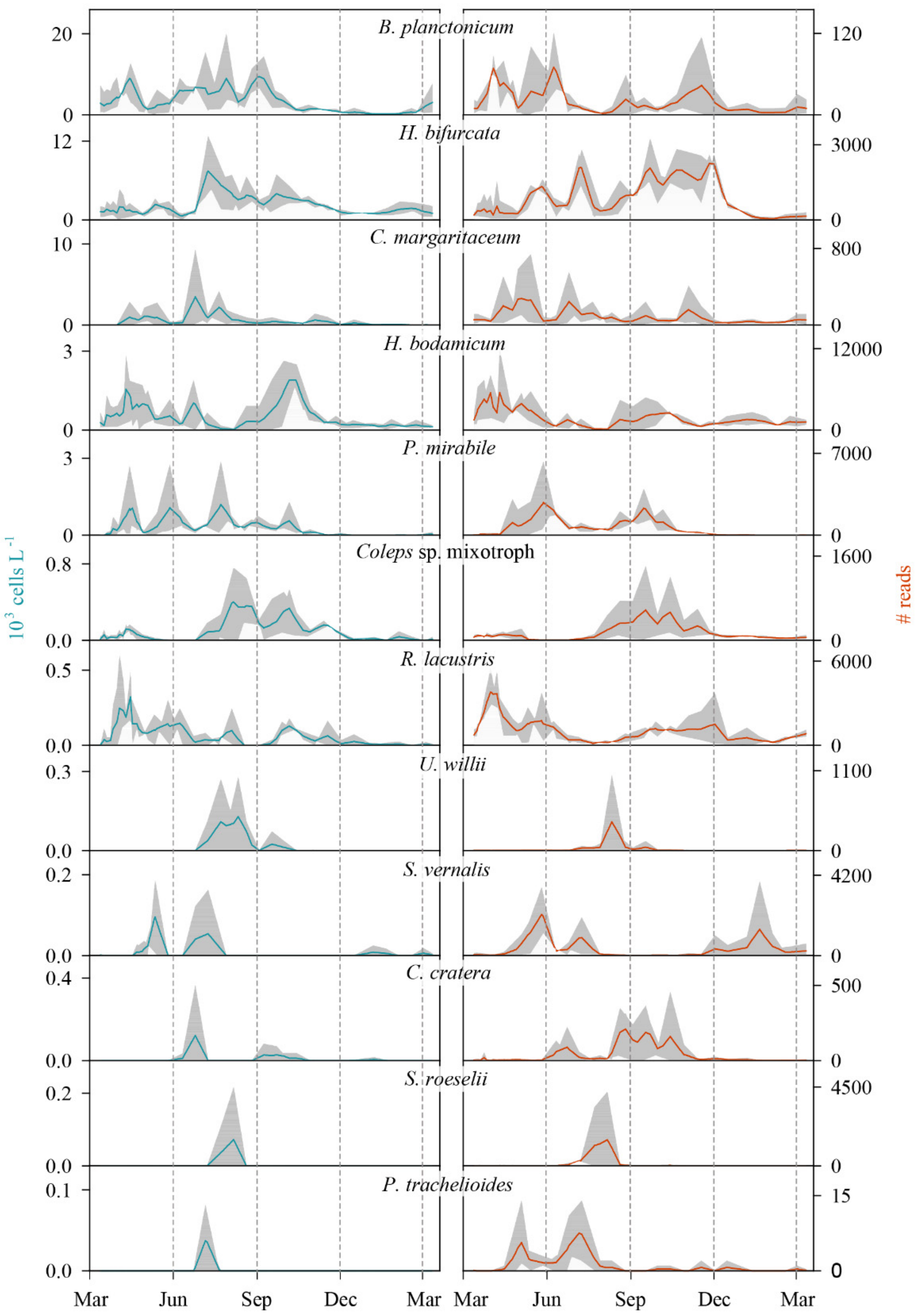

Supplementary Figure S3 Annual successions of the twelve selected ciliate species analyzed by morphotype counting (left panels) and HTS (right panels) from 5 m depth in Lake Zurich. First, each of the three annual datasets (see Figure 1, total n = 74 samples for each species and method) was interpolated on a daily base of Julian days. Afterwards, the three years were combined in one annual cycle showing the three years range of observations (grey shaded area) and the average (lines). The order of species reflects their average (n = 74) abundance based on counting, with the most abundant species on top and the rarest representative on bottom.

Supplementary Table S1 Average and maximum abundance (cells L^{-1}) and biomass (µg L^{-1}) values of ciliate morphospecies detected in Lake Zurich in 5 m depth from March 2014 to March 2017 ($n = 74$). The 48 clearly definable ciliate morphospecies were identical to described species or genera and sometimes comprised two or more species. The twelve selected species in this study are highlighted in bold.

	**Abundance: cells L^{-1} Average (Maximum)**	**Biomass: µg L^{-1} Average (Maximum)[a]**
Intramacronucleata		
Colpodea		
Cyrtolophosis sp. mixotroph	495 (13,665)	
Litostomatea		
Askenasia acrostomia[b]	70 (504)	2.2 (16.1)
Askenasia chlorelligera[b]	82 (911)	1.6 (18.2)
Askenasia volvox[b]	101 (1,621)	3.5 (56.7)
Askenasia sp. ≤ 25µm[b]	421 (6,944)	
Belonophrya pelagica	6 (243)	0.1 (2.7)
Cyclotrichium viride[b]	7 (216)	3.5 (108)
Didinium sp.	1 (54)	
Lacrymaria sp.	2 (81)	
Lagynophrya sp.	58 (648)	
Lagynophrya sp. mixotroph	27 (748)	
Monodinium chlorelligerum	47 (1,296)	1.4 (38.9)
Monodinium sp.	4 (139)	
Paradileptus elephantinus	8 (341)	8.3 (341.1)
Pelagodileptus trachelioides	2 (81)	7.1 (364.6)
Pelagolacrymaria sp.	9 (216)	
Rhabdoaskenasia minima	191 (833)	1.9 (8.3)
Mesodiniea[b]		
Mesodinium sp.	64 (1,026)	
Nassophorea		
Obertrumia sp.	0 (17)	
Oligohymenophorea		
Cinetochilum margaritaceum	544 (9,236)	2.7 (46.2)
Cothurnia annulata	143 (979)	2 (13.7)
Cyclidium spp.	2,084 (13,935)	
Cyclidium glaucoma		
unknown similar species		
Epistylis spp.	138 (1,414)	
Epistylis procumbens		
Epistylis anastatica		
Epistylis pygmaeum		

Supplementary Table S1 Continued

	Abundance: cells L^{-1} Average (Maximum)	Biomass: µg L^{-1} Average (Maximum) [a]
Intramacronucleata		
Oligohymenophorea		
Histiobalantium bodamicum	527 (2,809)	17.9 (95.5)
Pelagovorticella natans	44 (425)	4 (38.3)
Pseudohaplocaulus sp.	3 (162)	
Stokesia vernalis	9 (185)	3.6 (74.1)
Vorticella aquadulcis	95 (2,309)	1.4 (34.7)
Vorticella chlorellata	65 (1,620)	1.6 (40.5)
Vorticella vernalis	23 (1,368)	0.9 (54.7)
Phyllopharyngea		
Chilodonella sp.	22 (1,276)	
Prostomatea		
Balanion planctonicum[b]	3,446 (19,930)	6.9 (39.9)
***Coleps* sp. mixotroph**	102 (756)	6.1 (45.4)[d]
Urotricha spp.	4,579 (17,913)	
Urotricha farcta		
Urotricha furcata		
Urotricha pelagica		
unknown *Urotricha* spp.		
Spirotrichea		
Codonella cratera	9 (365)	0.2 (7.3)
Halteria bifurcata[c]	2,228 (12,726)	17.8 (101.8)
Limnostrombidium pelagicum	263 (2,485)	7.9 (74.6)
Limnostrombidium viride	2 (81)	0.1 (4.1)
Membranicola tamari	14 (162)	0.7 (8.1)
Pelagostrombidium fallax	5 (108)	0.6 (13)
Pelagostrombidium mirabile	298 (2,863)	8.9 (85.9)
Pelagohalteria viridis	867 (8,875)	6.9 (71)
Rimostrombidium lacustris	58 (378)	5.2 (34)
Rimostrombidium hyalinum/brachykinetum	337 (6,319)	
Rimostrombidium humile	2,548 (16,292)	10.2 (65.2)
Tintinnid spp.	545 (6,076)	
Tintinnidium pusillum		
Tintinnopsis cylindrata		
Uroleptus willii	14 (278)	1 (20)[e]
Postciliodesmatophora		
Heterotrichea		
Stentor roeselii	4 (216)	21.9 (1,080)

a) *Incertae sedis*, Gao et al. (2016) and Liu et al. (2015), respectively.

b) Biomass values only calculated when identification to species level was possible.

c) Possibly including *Halteria grandinella.*

d) Biomass value from *Coleps spetai.*

e) Own calculation (*U. willii* average cell size 120 x 38 µm. Formula to calculate cell volume: $V = 2/15 \times \pi \times 38\ \mu m \times 120\ \mu m$).

Supplementary Table S2 Average and standard error (SE), minimum and maximum values of number of reads for the 12 selected ciliates species (n = 74 for each species), which were extracted from our Lake Zurich database (containing eukaryotic V9 OTU's) with 97% or 99% sequence similarity, respectively.

	**Number of reads with:**					
	**97% identity**			**99% identity**		
	Average (SE)	Min	Max	Average (SE)	Min	Max
Balanion planctonicum	19 (3)	0	120	-	-	-
Cinetochilum margaritaceum	88 (16)	0	737	88 (16)	0	736
Codonella cratera	42 (11)	0	456	41 (11)	0	456
Coleps sp. mixotroph	150 (29)	0	1,403	150 (29)	0	1,400
Halteria bifurcata	888 (100)	1	3,232	865 (97)	1	3,068
Histiobalantium bodamicum	1,758 (242)	27	11,823	1,696 (234)	27	11,496
Pelagodileptus trachelioides	1 (0)	0	14	1 (0)	0	12
Pelagostrombidium mirabile	650 (120)	0	6,283	648 (119)	0	6,258
Rimostrombidium lacustris	977 (121)	18	5,462	973 (120)	18	5,450
Stentor roeselii	127 (72)	0	4,215	125 (71)	0	4,146
Stokesia vernalis	370 (85)	0	3,856	370 (85)	0	3,850
Uroleptus willii	24 (14)	0	1,032	24 (14)	0	1,032
